# The New Jersey Bowel Preparation Scale: A More Objective and Detailed Scoring System for Screening Colonoscopies

**DOI:** 10.1155/2019/8319747

**Published:** 2019-03-06

**Authors:** Qasim Salimi, Thayer Nasereddin, Neel Patel, Reza Hashemipour, Augustine Tawadros, Zamir Brelvi, Sushil Ahlawat

**Affiliations:** ^1^Department of Medicine, Rutgers New Jersey Medical School, Newark, New Jersey, USA; ^2^Division of Gastroenterology, Rutgers New Jersey Medical School, Newark, New Jersey, USA

## Abstract

**Goals:**

The goal of this study was to develop an objective and detailed scoring system to assess the quality of bowel preparation.

**Background:**

The quality of bowel preparation impacts the success of the colonoscopy. We developed and compared a new bowel preparation scoring system, the New Jersey Bowel Preparation Scale (NJBPS), with existing systems that are limited by a lack of detail and objectivity in the Boston Bowel Preparation Scale (BBPS) and the Ottawa Bowel Preparation Scale (OBPS).

**Methods:**

This was a single-center, prospective, dual-observer study performed at Rutgers New Jersey Medical School University Hospital. Patients who were at medium risk for colorectal cancer and undergoing outpatient screening colonoscopy were enrolled in the study, and their bowel preparation was assessed separately by an attending and a fellow using each of the bowel preparation scoring systems.

**Results:**

98 patients were analyzed in the study, of which 59% were female. Most of the patient population was African American (65%) or Hispanic (25%). The average age of the patient was 60 years. Chi-squared analysis using SPSS software revealed intraclass correlation coefficient values between attending and fellow scores for each scale. The NJBPS had the highest value at 0.988, while the BBPS and OBPS had values of 0.883 and 0.894.

**Limitations:**

Single-center study.

**Conclusions:**

The NJBPS and BBPS scores demonstrated a statistically significant agreement with each other. Overall, there was good interobserver agreement for all three scoring systems when comparing attendings to fellows for the same scoring system. However, the NJBPS possessed a stronger correlation.

## 1. Introduction

Colorectal cancer is the third leading cause of death in the United States. The American Cancer Society projects that there will be 140,000 new cases of colorectal cancers (CRC) diagnosed in 2018 alone. With improvements in preventive medicine strategies, the annual incidence of colon and rectal cancers has been reduced by 3.8% and 3.5%, respectively, in patients 55 years and older. However, in the cohort less than 55 years, the annual incidence of colon and rectal cancers has increased by 1.4 and 2.4% [1]. Thus, there is a continued need to refine the techniques used to detect cancerous and precancerous lesions through colonoscopy. The quality of bowel preparation directly impacts the success of a colonoscopy in detecting these lesions. Optimal visualization of the colonic mucosa is necessary to avoid missing lesions and repeating colonoscopy at an earlier interval [2, 3].

The American Society for Gastrointestinal Endoscopy (ASGE) and the American College of Gastroenterology (ACG) Taskforce on Quality in Endoscopy published quality indicators to measure the performance of colonoscopy. One of the quality indicators is the bowel preparation. To describe the quality of preparation, they proposed the use of terms such as “excellent,” “good,” “fair,” and “poor,” despite the lack of standardized definitions [4]. However, it is important to utilize a bowel-preparation scale that has been validated prospectively [5–7]. The two most common scales used for assessing bowel preparation is the Boston Bowel Preparation Scale (BBPS) and the Ottawa Bowel Preparation Scale (OBPS). They are bowel cleanliness rating scales originally designed and validated for use during colonoscopy-oriented research but have shown to be cumbersome and impractical for daily use and in routine clinical practice [5, 6, 8]. Therefore, there is a need for a scale that is both prospectively validated in an objective manner and easy to use on a daily basis.

To overcome these limitations, we developed and compared a new bowel preparation scoring system, the New Jersey Bowel Preparation Scale (NJBPS), with existing systems, the BBPS and the OBPS. Although the existing scoring systems moved away from subjective terms such as “excellent,” “good,” “fair,” and “poor,” by introducing a four-point scoring system, there still remains subjectivity in assigning each score. The novel New Jersey Bowel Preparation Scale is aimed at providing a more practical, reliable, and objective scoring system to be used with certainty by all providers. We envision that this rating scale could be used in clinical and research settings and for establishing guidelines on appropriate screening and surveillance intervals inclusive of bowel preparation quality.

## 2. Materials and Methods

The study was approved by the Rutgers New Jersey Medical School institutional review board in January 2017. This was a single-center, prospective, dual-observer study. Average-risk participants were enrolled on the day of their screening colonoscopy at Rutgers New Jersey Medical School University Hospital in Newark, New Jersey. Inclusion criteria included patients over the age of 50 years (African Americans ≥45 years old) that required screening colonoscopies. Exclusion criteria included patients ≥75 years old, personal history of adenomatous polyps or CRC, genetic syndrome predisposing to CRC, first-degree relatives with CRC or advanced adenoma, inflammatory bowel disease, or history of abdominal radiation therapy. All ethnicities as well as both sexes were eligible to participate. Enrollment and informed consent were limited to only English-speaking patients. A gastroenterology attending and fellow separately completed the scoring assessments for NJBPS, BBPS, and OBPS after each colonoscopy concluded. The scoring assessment for the NJBPS is described below in detail with a sample calculation included (Table 1). Scores were given for each segment of the colon (left colon, transverse colon, and right colon) after thorough washing and suctioning of stool. The average score of the three segment scores was the total score. If more than 500 mL liquid stool/fluid was removed during the washing/suctioning portions of the procedure, a penalty score of 15 was added to the total score. Instead of using basic numerical values like 0, 1, and 2 to score the segments, we chose 0, 25, and 50 to make the calculation more user-friendly and practical, avoiding score ranges that would include decimals essentially. It is also important to note that our recommendations for repeat colonoscopy, as described in Table 1, are based on the assumption that no clinically significant polyps or cancers are found.

A *t*-test analysis was done to compare significant differences between the mean male and female scores for each bowel preparation scale. This analysis was also used to compare any significant differences in ethnicity or insurance status.

We calculated the intraclass correlation coefficient (ICC) between attending and fellow for each scoring system in order to assess interobserver reliability. Testing was performed using a two-way mixed effects model with a 95% confidence interval. For ICC values, 0.75 to 1.00 is considered excellent, 0.60 to 0.74 is considered good, 0.40 to 0.59 is considered fair, and less than 0.40 is considered poor. These are measures of each scoring system's ability to reliably produce the same score with different observers [9]. To assess interobserver reliability, we calculated weighted kappa measures [10].

In order to compare scores between the NJBPS and BBPS, we first standardized the results of each scoring system into categorical outcomes. A NJBPS score of 0-30 was counted as “Good/Fair” while a score of 30-65 was counted as “Mediocre/Poor”. Similarly, a BBPS score of 5-9 was counted as “Good/Fair” while a score of 0-4 was counted as “Mediocre/Poor”. The cutoff of 5 for the BBPS was chosen on the basis of the best adenoma detection rates (40% vs 24%) for scores that were ≥5 [8]. We then utilized a Pearson's chi-squared test comparing the NJBPS and BBPS categorical test outcomes to see if there was a significant difference. All statistical analysis was done using IBM SPSS software version 25. A *p* value of <0.05 represents statistical significance.

## 3. Results

A total of 113 patients undergoing screening colonoscopy were initially enrolled in this study. Due to incomplete scoring assessments by attending, fellow, or both, 15 patients were excluded from the study. The final number of enrolled patients who had complete scoring assessments was 98, of which 59% were female and 41% were male. The patient population consisted of more African American (65%) and Hispanic (25%) patients compared to Caucasian patients (2%). The average patient was 60 years old, and most of the patients had insurance (83%) (Table 2).

The *t*-test analysis for equality of means showed a significant difference between male and female average scores for NJBPS and OBPS attending scores [NJBPS, *p* = 0.012; OBPS, *p* = 0.004]. Overall, females averaged a lower score when compared to males [scale, female vs. male]: [NJBPS, 15.19 vs. 22.25] and [OBPS, 4.81 vs. 6.13]. The BBPS had no significant difference between male and female average scores (Table 3).

Attending vs. fellow mean scores for each scoring system were analyzed to measure interobserver agreement using the kappa ICC. The NJBPS exhibited a superior interobserver agreement, ICC = 0.99: 95% CI [0.98, 0.99]. The BBPS and OBPS also exhibited excellent interobserver agreement, ICC = 0.88: 95% CI [0.83, 0.92] and ICC = 0.89: 95% CI [0.84, 0.93], respectively (Table 4).

The attending scores from NJBPS and BBPS were categorized between “Good/Fair” and “Mediocre/Poor” to assess for any correlation between the two scales. A score of 0-30 from NJBPS cross-tabulated with a score of >5 from BBPS score and was categorized as “Good/Fair.” A score of >30 from NJBPS cross-tabulated with a score <5 and was categorized as “Mediocre/Poor.” Pearson's chi-squared analysis showed a significant correlation between NJBPS and BBPS scoring (*χ*^2^ = 48.034, *p* = .0001) (Table 5).

## 4. Discussion

To overcome the limitations of current bowel preparation scoring systems, we developed and compared a new scoring system, the New Jersey Bowel Preparation Scale (NJBPS) that is more objective and reliable. Overall, there was good interobserver agreement for all three scoring systems when comparing attendings to fellows for the same scoring system. However, the New Jersey Bowel Preparation Scale (NJBPS) intraclass correlation coefficient (ICC) was higher than those of the Boston Bowel Preparation Scale (BBPS) and the Ottawa Bowel Preparation Scale (OBPS), demonstrating that this system may have more practical utility in daily analysis and be more reliable. The ICC value of 0.988 for the NJBPS indicates that the correlation between attending and fellow scores is very strong. The NJBPS identifies that the entirety of the colon is not equally prepared for colonoscopy, allowing the various scores to be assigned to each of the three segments of the colon (ascending, transverse, and descending). It has been shown that the use of a three colonic segment scoring system is best for polyp detection and increases interobserver reliability [5, 8, 11]. This may be a reflection of an easy to understand and interpret scoring system, making it more reliable as a result. In addition, giving values of 0, 25, and 50 for each of the three segments and having an average score is a more accurate depiction of bowel cleanliness. Moreover, the numerical aspiration penalty in our scoring system adds an objective element not seen in the other systems, as we quantify the amount of liquid stool suctioned out and assign a penalty score if the aspirate volume is greater than 500 mL. The chi-squared analysis showed that there was a statistically significant agreement between NJBPS and BBPS scores. This finding shows that these scores are more likely to significantly agree than disagree, indicating good correlation between scoring systems. The Boston Bowel Preparation Scale ICC was similar to that of the 2014 study, indicating good reliability between studies [8].

Multiple bowel rating scales that were previously published were mainly designed to compare the efficacy of two or more bowel preparation methods [12–16]. This study distinguishes itself by comparing multiple bowel preparation scoring systems. The NJBPS scoring system is also designed to be more objective which can be better suited for colonoscopy outcomes research, such as studies aimed at defining appropriate screening and surveillance intervals that are determined by bowel preparation quality. It can also be used when comparing bowel preparations, where the clinical effectiveness of the preparations can be tested.

There were no significant differences when comparing most secondary outcomes. However, female patients had overall better bowel preparation mean scores. This was statistically significant for NJBPS and OBPS scores, but not for BBPS scores. In the New Jersey Bowel Preparation Scale, female patients had lower average scores when compared to male patients (15/“Good” vs. 22/“Fair”). This finding has been shown in previous studies, where male gender was a predictor of inadequate colon preparation [17]. However, there is a 2002 study that shows more females when compared to males (75 vs. 69) had an incomplete colonoscopy due to a poor bowel preparation, but it is unclear if this was statistically significant [18]. There were also significantly worse bowel preparations in Caucasians when compared to Black/Hispanic/Other populations. This finding was also observed in NJBPS and OBPS, but the results were nonsignificant. There were only two Caucasian patients enrolled in our study; given that our university hospital is located in an urban area, this may have skewed the results—a study with a more prevalent Caucasian population is needed to truly compare.

We believe that the NJBPS has reasonable validity for general use in research studies. Our study has several strengths including assessment of the interobserver reliability as well as correlation analysis between attending scores among three different bowel preparation scales. We also understand our novel scale's certain limitations. The NJBPS requires assessment of bowel cleanliness of the ascending, transverse and left colon segments, and landmarks distinguishing these segments may be poorly defined or difficult to recognize. While this is not limited to NJBPS, it would be reasonable for future studies to observe the interrater reliabilities for various segments among endoscopists. It is encouraging that we found similar results among the fellows and attendings suggesting that the NJBPS can be used by physicians of various levels of experience. However, our study was limited to a single, urban, academic institution that serves a diverse and underserved, predominantly Black and Hispanic population, potentially limiting the generalizability of our results. Despite also being significantly correlated with the BBPS, future studies with the NJBPS should focus on adenoma detection rates. Also NJBPS was not evaluated for use during other bowel imaging procedures, such as CT colonography or capsule endoscopy. Thus, it is not clear that NJBPS can be effectively used in noncolonoscopy bowel imaging.

In conclusion, there was good interobserver agreement for all three scoring systems when comparing attendings and fellows; this also correlated to the similar secondary outcomes when looking at the fellow scores. Therefore, the New Jersey Bowel Preparation Scale is a user-friendly, reliable, and more objective way to analyze the quality of bowel preparation and its scores correlate to the Boston Bowel Preparation Scale. Further studies can be done in the future to analyze potential differences in adenoma detection rate and to apply the scale in other environments (i.e., suburban, rural, etc.).

## Figures and Tables

**(a):** 

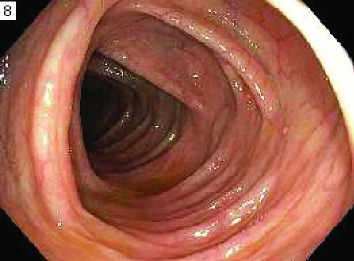	**0**—residual material present in less than 1 quadrant of the image or completely clear. This represents the best (most clean) score for a given segment.
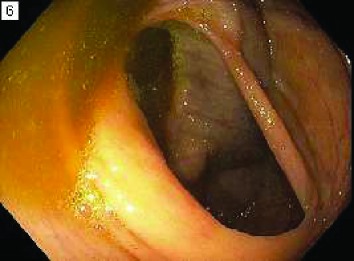	**25**—residual material present in at least 1 but less than 2 quadrants. No solid stool is present in any quadrant.
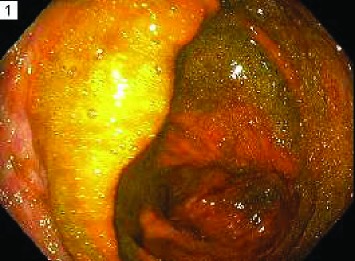	**50**—residual material present in at least 2 quadrants or solid stool in any quadrant. This represents the worst (least clean) score for a given segment.

**(b):** 

Total score: average segment score [(left colon + transverse colon + right colon)/3] Penalty score: add 15 to total score if aspirate volume more than 500 cc		
Total score	Description	Recommendation for repeat colonoscopy
0-15	Good	Follow guidelines
15-30	Fair	3-5 years
30-45	Mediocre	1-3 years
>45	Poor	1 year or as soon as possible

Example score: total score: (left colon score: 50 + transverse colon score: 25 + right colon score: 0)/3 = 25, penalty score: 330 cc aspirate, no penalty score needs to be added, total score = 25, which equates to fair bowel prep, with recommendation for repeat, colonoscopy in 3-5 years.

**Table 2:** Patient demographics.

	*n*	Percentage (%)	Mean age^∗^	Std dev±
Age	98	—	59.5	8.2
Gender				
Female	58	59.2		
Male	40	40.8		
Ethnicity				
Black	64	65.3		
Hispanic	24	24.5		
Caucasian	2	2.0		
Unknown/other	8	8.2		
Insurance				
Yes	81	82.7		
None	4	4.1		
Charity care	13	13.3		

^∗^Age in years. ± means standard deviation.

**Table 3:** Mean scores and equality of means of attending scores.

Scale	Gender	*n*	Mean	*t*-test^∗^	*p* value
NJBPS	Male	40	22.25	2.56	0.012
	Female	58	15.19		
BBPS	Male	40	5.13	-1.66	0.100
	Female	58	5.74		
OBPS	Male	40	6.13	2.91	0.004
	Female	58	4.81		

^∗^ *t*-test for equality of means.

**Table 4:** Intraclass correlation coefficient of attending vs fellow by different bowel preparation scoring systems.

Scale	NJBPS	BBPS	OBPS
Kappa ICC	0.99 95% CI [0.98, 0.99]	0.88 95% CI [0.83, 0.92]	0.89 95% CI [0.84, 0.93]

*ICC*: intraclass correlation coefficient; CI: confidence interval.

**Table 5:** Cross-tabulation of NJBPS and BBPS with chi-square analysis.

			BBPS attending		Total	*p* value
			Good/Fair	Mediocre/Poor		
NJBPS attending	Good/Fair	Count	73	8	81	**p** = 0.0001
	Mediocre/Poor	Count	2	15	17	
		Total	75	23	98	

## Data Availability

All data used to support the findings of this study are available from the corresponding author upon request. This excludes data that is restricted in order to protect patient privacy and only limited to researchers who meet the criteria for access to confidential data.
